# Lipoprotein(a), smoking, and diabetes are additive but not interactive risk factors for major adverse cardiovascular events after acute coronary syndrome

**DOI:** 10.1016/j.ajpc.2026.101747

**Published:** 2026-08-31

**Authors:** Gregory G. Schwartz, Michael Szarek, Vera A. Bittner, Deepak L. Bhatt, Rafael Diaz, Shaun G. Goodman, Robert A. Harrington, Ghada Hassan, J. Wouter Jukema, Irena Stevanovic, Hagai Tavori, Samantha Thielen, Harvey D. White, P. Gabriel Steg

**Affiliations:** a Division of Cardiology, University of Colorado School of Medicine, Aurora, CO, USA; b Division of Cardiovascular Disease, University of Alabama Birmingham, Birmingham, AL, USA; c Mount Sinai Fuster Heart Hospital, Icahn School of Medicine at Mount Sinai, New York, NY, USA; d Estudios Cardiologicos Latinoamerica, Rosario, Argentina; e St Michael's Hospital and Canadian VIGOUR Centre, Toronto, Ontario, and Edmonton, Alberta Canada; f Weill Cornell Medicine, New York, NY, USA; g Sanofi, Helsinki, Finland; h Leiden University Medical Center, Leiden, and Netherlands Heart Institute, Utrecht, the Netherlands; i Green Lane Cardiovascular Research Unit, University of Auckland, Auckland, New Zealand; j Université Paris-Cité, AP-HP, Hôpital Bichat, INSERM-UMR1148, and FACT (French Alliance for Cardiovascular Trials), Paris, France; k Sanofi, Yakum, Israel

**Keywords:** Lipoprotein(a), Smoking, Diabetes, Cardiovascular events, Acute coronary syndrome, Alirocumab

Lipoprotein(a) is a genetically validated, independent risk factor for myocardial infarction, ischemic stroke, peripheral artery disease, and need for arterial revascularization procedures. This is believed to be due to a combination of atherogenic, inflammatory, and pro-thrombotic effects of elevated lipoprotein(a) [1].

The acquired, common factors of smoking and diabetes mellitus are also thought to convey cardiovascular risk through a similar triad of atherogenic, inflammatory, and pro-thrombotic mechanisms [2,3]. Whether lipoprotein(a), smoking, and diabetes are additive or interactive (synergistic) risk factors is unknown and might influence the urgency and intensity of risk factor management.

We addressed this question in a post hoc analysis of the ODYSSEY OUTCOMES trial (clinicaltrials.gov NCT01663402), an international randomized, double-blind, placebo-controlled evaluation of the effects of the proprotein convertase subtilisin/kexin type 9 (PCSK9) inhibitor, alirocumab, on major adverse cardiovascular events (MACE) in 18,924 patients with recent acute coronary syndrome and elevated atherogenic lipoproteins despite high-intensity or maximum tolerated statin treatment [4]. All participants provided written informed consent. The trial was approved by the institutional review board or ethics committee at each site. In previous univariate analysis, elevated lipoprotein(a) increased the risk of the primary MACE endpoint (coronary heart disease death, non-fatal myocardial infarction, ischemic stroke, and hospitalization for unstable angina) and ischemia-driven coronary revascularization in the placebo group and was associated with larger absolute and relative reductions in that risk with alirocumab [5]. Similarly, diabetes was associated with the risk of MACE in the placebo group and with absolute reduction in that risk with alirocumab [6]. An association of smoking with the risk of MACE in the placebo group and risk reduction with alirocumab has not been investigated previously.

In the present analysis, we determined the 4-year Kaplan Meier incidence of MACE in the placebo group and the reduction in MACE by alirocumab according to the presence or absence of these three risk factors, each considered dichotomously: lipoprotein(a) at its median value, prevalent diabetes, and smoking (current vs previous or never) at the time of randomization. We examined an expanded MACE endpoint (comprising all cardiovascular deaths and ischemia-driven coronary revascularization in addition to non-fatal myocardial infarction, ischemic stroke, and hospitalization for unstable angina) since each component was directly associated with lipoprotein(a) levels and/or reduced by alirocumab. Cox regression models adjusted for age, sex, geographic region, low density lipoprotein cholesterol (LDL-C), and systolic blood pressure were used to evaluate the association of Lp(a) quartile with MACE, without or with further adjustment for smoking and diabetes. Lipoprotein(a) was measured in mass concentration by an immunoturbidometric assay with rabbit polyclonal anti-Lp(a) detection antibody and inter-assay coefficient of variation 3.1–4.8% depending on Lp(a) concentration (Siemens, Healthcare Diagnostics, Malvern, PA, USA).

Baseline characteristics of the ODYSSEY OUTCOMES trial population have been previously published [4] and include mean age 58.5 years; 25.2% female sex; and enrollment in Central or Eastern Europe (28.7%), Western Europe (22.0%), Canada or United States (15.2%), Latin America (13.7%), Asia (12.1%), or other parts of the world (8.3%). The prevalence of diabetes [6] was 28.8% (of whom 99.3% had type 2 and 0.7% had type 1 diabetes) and 24.1% were active smokers at the time of enrollment. Mean systolic blood pressure was 128 mmHg. Median (quartile [Q] 1, Q3) LDL-C and lipoprotein(a) were 87 (73, 104) and 21.2 (7, 60) mg/dL, respectively.

Patients were followed for median (Q1, Q3) 2.8 (2.3, 3.4) years. The 4-year Kaplan Meier incidence of the expanded MACE endpoint was 19.7% (1474 events) in the placebo group and 17.4% (1301 events) in the alirocumab group (hazard ratio [HR] 0.87; 95% confidence interval [CI] 0.81, 0.94).

In the placebo group (n = 9462), Lp(a) ≥median was associated with greater risk of MACE than Lp(a) <median (21.3% versus 18.1%, P < 0.0001). In both Lp(a) categories, risk of MACE increased when active smoking, diabetes, or both were present (Fig. 1 **Part A**). In Cox regressionmodels without adjustment for either smoking or diabetes, Lp(a) quartile was associated with MACE (HR Q4 vs Q1 1.26; 95% CI 1.09, 1.46; Lp(a) quartile P_trend_=0.0006). In a model incorporating smoking, Lp(a) quartile remained associated with risk of MACE (HR Lp(a) Q4 vs Q1 1.27; 95% CI 1.10, 1.47; Lp(a) quartile P_trend_=0.0004) while smoking was an independent risk factor (HR 1.23; 95% CI 1.09, 1.39; P = 0.0007), but without Lp(a) x smoking interaction (P_interaction_=0.86). Similarly, in a model incorporating diabetes, Lp(a) quartile remained associated with risk of MACE (HR Lp(a) Q4 vs Q1 1.29; 95% CI 1.12, 1.49; Lp(a) quartile P_trend_=0.0002) while diabetes was an independent risk factor (HR 1.63; 95% CI 1.47, 1.81; P < 0.0001), but without Lp(a) x diabetes interaction (P_interaction_=0.31). Finally, when adjusted for both diabetes and smoking Lp(a) remained a significant predictor of MACE (HR Lp(a) Q4 vs Q1 1.30; 95% CI 1.13, 1.51; Lp(a) quartile P_trend_=0.0001) while both smoking (HR 1.25; 95% CI 1.11, 1.41; P = 0.0003) and diabetes (HR 1.64; 95% CI 1.47, 1.82; P < 0.0001) remained independent predictors of risk. There was no 3-way interaction on MACE among Lp(a), smoking, and diabetes (P_interaction_=0.28).

**Fig. 1:**
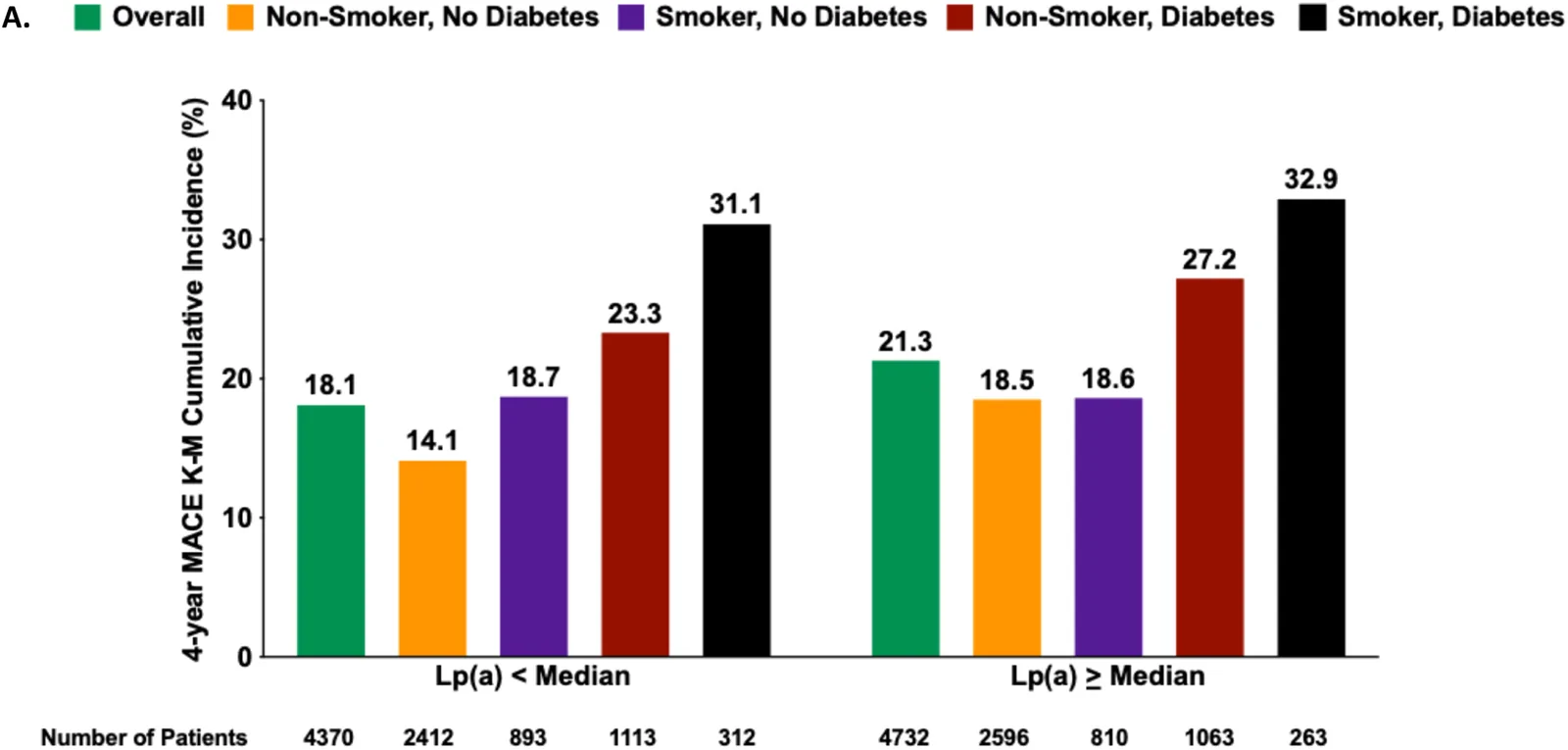

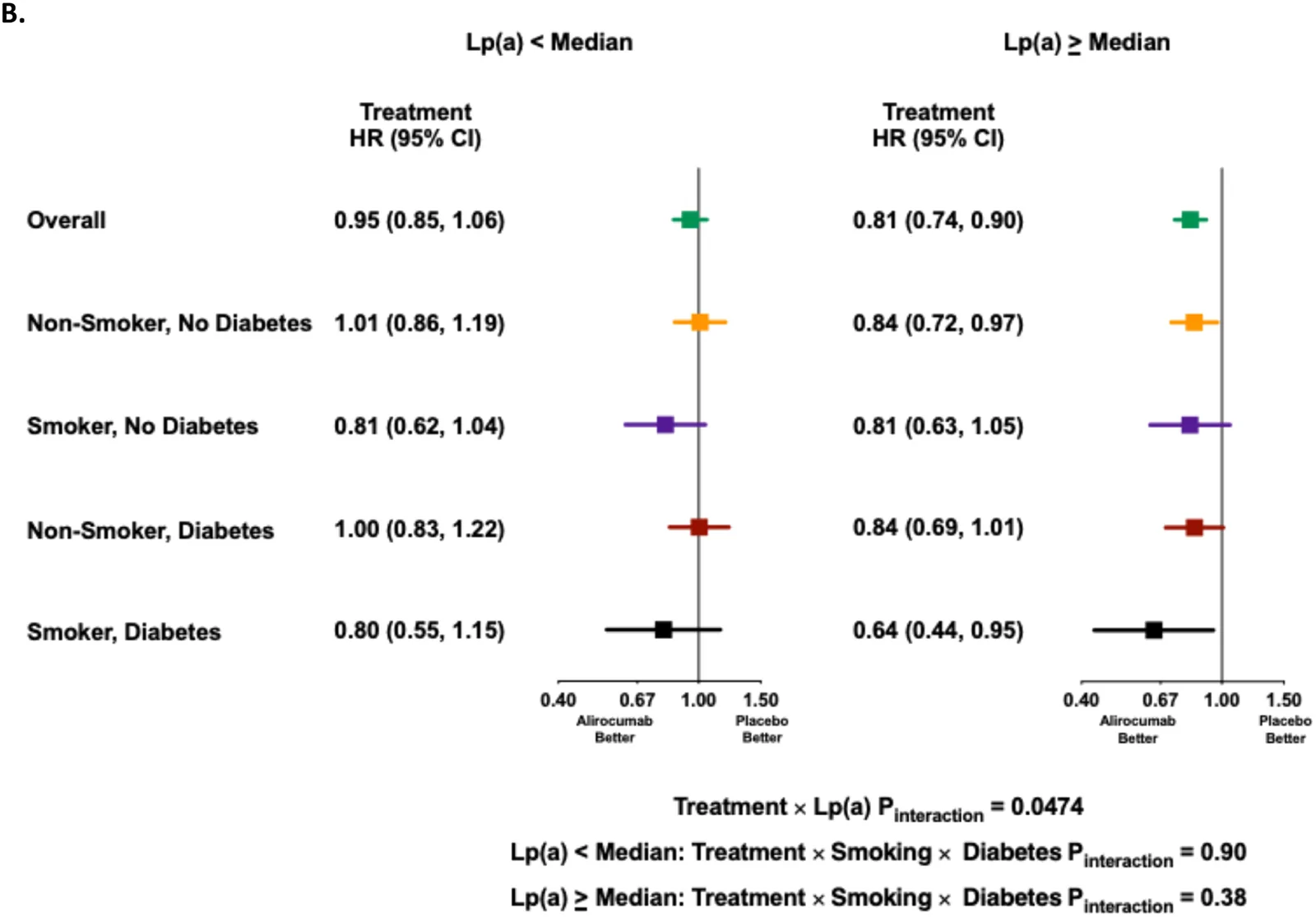
**A:** 4-year Kaplan Meier cumulative incidence of major adverse cardiovascular events (MACE) in the placebo group of the ODYSSEY OUTCOMES trial (n = 9462) dichtomized at the median baseline lipoprotein(a) [Lp(a)] concentration (21.6 mg/dL). In each Lp(a) category, MACE incidence is shown overall and in subcategories defined by active smoking or diabetes at baseline. The components of MACE are defined in the text. **B:** Treatment hazard ratio (HR) for MACE (alirocumab/placebo) with 95% confidence intervals are shown for baseline Lp(a) categories less than, or at least the median aggregate baseline concentration of both treatment groups, 21.2 mg/dL. Within each Lp(a) category, treatment HR is shown overall and according to the presence of smoking, diabetes, or both. For patients with Lp(a) < and ≥median, respectively, n = 9543 and 9381 (overall), 4869 and 5147 (non-smoker, no diabetes), 1833 and 1631 (smoker, no diabetes), 2237 and 2111 (non-smoker, diabetes), and 604 and 492 (smoker, diabetes).

The treatment HR for MACE (alirocumab/placebo) was evaluated according to the presence of the 3 dichotomous risk factors (Fig. 1 **Part B**). When dichotomized at median Lp(a), treatment HR was more robust with Lp(a) ≥median (P_interaction_ = 0.047). In each combination of Lp(a) category with smoking or diabetes, the treatment HR was numerically more robust in the Lp(a) ≥median category. However, within each Lp(a) category there was no heterogeneity of the treatment HR according to the presence of smoking or diabetes.

Limitations of this analysis include its post hoc, and thus exploratory nature. An unbiased analysis of interaction between Lp(a) and LDL-C was not possible since patients in ODYSSEY OUTCOMES were selected for elevated LDL-C (92.1% with LDL-C ≥ 70 mg/dL on optimized statin treatment). Similarly, we did not explore interaction of Lp(a) and hypertension since approximately two thirds of trial participants had a history of hypertension [4]. However, the adjustment for baseline LDL-C and systolic blood pressure had minimal effect on the reported relationships (data not shown). Smoking status during the trial was not documented.

In conclusion, Lp(a), active smoking, and diabetes, each believed to convey cardiovascular risk through atherogenic, inflammatory, and thrombogenic effects, were found to be additive, but not interactive risk factors for MACE in patients with recent ACS receiving optimized statin therapy. Lp(a) remained an independent predictor of MACE after adjustment for smoking, diabetes, or both, and LDL-C and systolic blood pressure. Although the treatment HR for MACE (alirocumab/placebo) was more robust when Lp(a) is ≥21 mg/dL, compared with <21 mg/dL, within each Lp(a) category there was no heterogeneity of treatment HR according to the presence of smoking, diabetes, or both. The findings indicate that in patients with recent acute coronary syndrome on optimized statin therapy, elevated Lp(a), smoking, and diabetes remain incremental risk factors and may require individualized preventive measures.

## Funding

The ODYSSEY OUTCOMES trial was funded by Sanofi and Regeneron Pharmaceuticals.

## CRediT authorship contribution statement

**Gregory G. Schwartz:** Conceptualization, Funding acquisition, Investigation, Supervision, Writing – original draft. **Michael Szarek:** Data curation, Formal analysis. **Vera A. Bittner:** Investigation, Writing – review & editing. **Deepak L. Bhatt:** Investigation, Writing – review & editing. **Rafael Diaz:** Investigation, Writing – review & editing. **Shaun G. Goodman:** Investigation, Writing – review & editing. **Robert A. Harrington:** Investigation, Writing – review & editing. **Ghada Hassan:** Investigation, Writing – review & editing. **J. Wouter Jukema:** Investigation, Writing – review & editing. **Irena Stevanovic:** Investigation, Writing – review & editing. **Hagai Tavori:** Investigation, Writing – review & editing. **Samantha Thielen:** Investigation, Writing – review & editing. **Harvey D. White:** Investigation, Writing – review & editing. **P. Gabriel Steg:** Investigation, Writing – review & editing.

## Declaration of competing interest

The authors declare the following financial interests/personal relationships which may be considered as potential competing interests: **Gregory Schwartz** reports research support from the US Department of Veterans Affairs, research support to the University of Colorado from AstraZeneca, Sanofi and Silence Therapeutics, and support for travel to trial meetings from the University of Oxford. **Michael Szarek** reports receives salary support from CPC, a non-profit academic research organization affiliated with the University of Colorado, that receives or has received research grant/consulting funding between July 2021 and July 2026 from the following organizations: 35Pharma, Inc, Abbott Laboratories, Agios Pharmaceuticals, Inc., Alexion Pharma Godo Kaisha, American Heart Association, American Journal Managed Care, Amgen Inc., Amgen USA, Inc., Anthos Therapeutics, Inc., Arrowhead Pharmaceuticals, AstraZeneca Pharma India, AstraZeneca Pharmaceuticals LP, AstraZeneca UK Ltd, Autonomy Bio, Inc, Bayer, Bayer Aktiengesellschaft, Beth Israel Deaconess Medical Center, Better Therapeutics, Boston Clinical Research Institute, LLC, Bristol-Myers Squibb, Cleerly, Inc., Clergy United for the Transformation of Sandtown, Colorado Dept of Public Health and Environment, Congress Inc, Cook Regentec LLC, Eidos Therapeutics, Inc., EluraBio, Inc, Esperion Therapeutics, Inc, Faraday Pharmaceuticals, Inc., Gasherbrum Bio Inc, Insmed, IsomAb Limited, JanOne Biotech Holdings, Inc., Janssen Global Services, Janssen Pharmaceuticals, Inc, Janssen Scientific Affairs LLC, Las Animas and Huerfano Counties, District Health Dept, Lexicon Pharmaceuticals, Inc., Lilly USA, LLC, Medison Pharma, Medpace, Inc., Merck Sharp & Dohme Corp., Nectero Medical, Inc, NewAmsterdam Pharma, Novartis Pharmaceuticals Corporation, Novo Nordisk Inc., Pfizer, Piper Sandler & Co, PPD Development, L.P., Prothena Biosciences Limited, Regeneron, Regents of the University of Colorado (aka UCD), Sanifit Therapeutics S.A., Sanofi, Silence Therapeutics PLC, Stanford University, Stealth BioTherapeutics Inc., The Brigham and Women's Hospital, Thrombosis Research Institute, Tourmaline Bio, Inc, University of Colorado, University of Pittsburgh, VarmX, Verve Therapeutics, WraSer, LLC, and reports serving as a consultant or research support (or both) from Amarin, Lexicon, NewAmsterdam, Novartis, Regeneron, Sanofi, Silence, and Tourmaline. **Deepak L. Bhatt** reports Advisory Boards: Angiowave, Antlia Bioscience, Bayer, Boehringer Ingelheim, CellProthera, Cereno Scientific, E-Star Biotech, High Enroll, Janssen, Level Ex, McKinsey, Medscape Cardiology, Merck, NirvaMed, Novo Nordisk, Repair Biotechnologies, Stasys, SandboxAQ (stock options), Tourmaline Bio, Viatris; Board of Directors: American Heart Association New York City, Angiowave (stock options), Bristol Myers Squibb (stock), DRS.LINQ (stock options), High Enroll (stock); Consultant: Alnylam, Altimmune, Broadview Ventures, Corcept Therapeutics, Corsera, GlaxoSmithKline, Hims, Sanofi, SERB, SFJ, Summa Therapeutics, Worldwide Clinical Trials; Data Monitoring Committees: Acesion Pharma, Assistance Publique-Hôpitaux de Paris, Baim Institute for Clinical Research, Boston Scientific (Chair, PEITHO trial), Cleveland Clinic, Contego Medical (Chair, PERFORMANCE 2), Duke Clinical Research Institute, Mayo Clinic, Mount Sinai School of Medicine (for the ABILITY-DM trial, funded by Concept Medical; for ALLAY-HF, funded by Alleviant Medical), Novartis, Population Health Research Institute; Rutgers University (for the NIH-funded MINT Trial); Honoraria: American College of Cardiology (Senior Associate Editor, Clinical Trials and News, ACC.org; Chair, ACC Accreditation Oversight Committee), Arnold and Porter law firm (work related to Sanofi/Bristol-Myers Squibb clopidogrel litigation), Baim Institute for Clinical Research (AEGIS-II executive committee funded by CSL Behring), Belvoir Publications (Editor in Chief, Harvard Heart Letter), Canadian Medical and Surgical Knowledge Translation Research Group (clinical trial steering committees), CSL Behring (AHA lecture), Duke Clinical Research Institute, Engage Health Media, HMP Global (Editor in Chief, Journal of Invasive Cardiology), Medtelligence/ReachMD (CME steering committees), MJH Life Sciences, Oakstone CME (Course Director, Comprehensive Review of Interventional Cardiology), Philips (Becker's Webinar on AI), Population Health Research Institute, WebMD (CME steering committees), Wiley (steering committee); Other: Clinical Cardiology (Deputy Editor, unpaid); Progress in Cardiovascular Diseases (Deputy Editor, unpaid); Added Health (Editorial Board; stock options); Patent: Sotagliflozin (named on a patent for sotagliflozin assigned to Brigham and Women's Hospital who assigned to Lexicon; neither I nor Brigham and Women's Hospital receive any income from this patent); Research Funding: Abbott, Acesion Pharma, Afimmune, Alnylam, Amarin, Amgen, AstraZeneca, Atricure, Bayer, Boehringer Ingelheim, Boston Scientific, CellProthera, Cereno Scientific, Chiesi, Cleerly, CSL Behring, Faraday Pharmaceuticals, Fractyl, Idorsia, Janssen, Javelin, Lexicon, Lilly, Medtronic, Merck, MiRUS, Moderna, Novartis, Novo Nordisk, Pfizer, PhaseBio, Regeneron, Reid Hoffman Foundation, Roche, Sanofi, Stasys, 89Bio; Royalties: Elsevier (Editor, Braunwald’s Heart Disease); Site Co-Investigator: Cleerly. **Vera A. Bittner** reports grant support from Sanofi, Regeneron Pharmaceuticals, Amgen, Astra Zeneca, DalCor, Esperion, and Novartis; consulting fees from Pfizer; honoraria from Medscape; and fees for data safety monitoring boards for the National Institutes of Health and for Verve Therapeutics. **Rafael Diaz** reports research grants from Sanofi, DalCor Pharmaceuticals, Population Health Research Institute, Duke Clinical Research Institute, the TIMI (Thrombolysis in Myocardial Infarction) group, Amgen, Cirius, Montreal Health Innovations Coordinating Center, and Lepetit; and personal fees, as a member of the Executive Steering Committee, from Amgen and Cirius. **Shaun G. Goodman** reports research grant support (e.g., steering committee or data and safety monitoring committee) and/or speaker/consulting honoraria (e.g., advisory boards) from: Alnylam, Amgen, AstraZeneca, Bayer, Boehringer Ingelheim, Bristol Myers Squibb, CSL Behring, CYTE Ltd., Eli Lilly, HLS Therapeutics, Merck, Novartis, Novo Nordisk A/C, Pendopharm/Pharmascience, Pfizer, Prolocor Inc., Regeneron, Roche, Sanofi, Tolmar Pharmaceuticals, Viatris; and salary support/honoraria from the Canadian Heart Research Centre and MD Primer, Canadian VIGOUR Centre, Cleveland Clinic Coordinating Centre for Clinical Research, Duke Clinical Research Institute, Jewish General Hospital\ CIUSSS Centre-Ouest-de-l'Ile-de-Montreal, New York University Clinical Coordinating Centre, Peter Munk Cardiac Centre Clinical Trials and Translation Unit, Ted Rogers Centre for Heart Research, TIMI Study Group (Brigham Health). **Robert A. Harrington** reports research relationships with Baim Institute (DSMB), Colorado Prevention Center (CPC) Clinical Research (DSMB), Edwards Lifesciences (RCT), Janssen (RCT), Merck/BWH (DSMB); Consulting relationships: Atropos Health, Basking Biosciences, Bitterroot Bio, BridgeBio Pharma, Corsera Health, Element Science, HeartBeam, Medscape/WebMD; Board of Directors: American Heart Association, Cytokinetics, Marea Therapeutics. **Ghada Hassan, Irena Stevanovic** and **Hagai Tavori** are employees of Sanofi. **J. Wouter Jukema** reports his department has received research grants from and/or was speaker (with or without lecture fees) on a.o. (CME accredited) meetings sponsored/supported by Abbott, Amarin, Amgen, Athera, Biotronik, Boston Scientific, Dalcor, Daiichi Sankyo, Edwards Lifesciences, GE Healthcare Johnson and Johnson, Lilly, Medtronic, Merck-Schering-Plough, Novartis, Novo Nordisk, Pfizer, Roche, Sanofi Aventis, Shockwave Medical, the Netherlands Heart Foundation, CardioVascular Research the Netherlands (CVON), the Netherlands Heart Institute and the European Community Framework KP7 Program. **Samantha Thielen** reports no competing financial interests or personal relationships. **Harvey D. White** reports grant support paid to the institution for the ODYSSEY OUTCOMES trial from Sanofi and Regeneron Pharmaceuticals, for the STRENGTH Trial from Omthera Pharmaceuticals, for the HEART-FID Study from American Regent, for the Dal GenE study from DalCor Pharma UK Inc, for the AEGIS II Study from CSL Behring, for the Clear Outcomes Study from Esperion Therapeutics, for the SOLIST-WHF and SCORED studies from Sanofi Aventis Australia Pty Ltd, for the Librexia AF and ACS studies from Janssen, and for ISCHEMIA and the MINT studies from the National Institutes of Health. He also received personal fees as a steering committee member for DalCor Pharma UK, CSL Behring, Sanofi Australia Pty Ltd, Janssen and Esperion Therapeutics. He was on advisory boards for CSL Behring and Genentech. **P. Gabriel Steg** reports grants, personal fees, and nonfinancial support from Sanofi; grants and personal fees from Amarin, Servier, and Bayer; and personal fees from Amgen, AstraZeneca, BMS, Boehringer Ingelheim, Idorsia, Pfizer, and Novartis.
